# 

**Published:** 1904-07-15

**Authors:** 


					﻿CONTENTS.
Extracts from the Proceedings of the Section on Stomat-
ology of the American Medical Association -	-	325
Practical Use of Nitrous Oxide,
By E. R. Jackson, D.D.S. 335
Permanency of the Teeth as seen from a Study of Com-
parative Dental Anatomy.
By Robt. B. Howell, D.D.S. 338
Esthetic Dentistry Required, - By IV. T. M' Lean 347
President’s Address, - By J. P. Beauman, D.D.S. 352
The Cuspid Teeth, -	- By A. O. Hunt, D.D.S. 356
UITORIAL’ -	- T LIBRARY T 338
Corrbspondkncb, - SURGE O'N GENt RAI'S 0F FlCE J
Announcements,	-	-	-	-	-	-	-	361
Bibliography,- -	-	,	366
Obituary, ---------	369
Indents, -	-	-	-	-	-	-	-	371
				

